# A Complex Molecular Interplay of Auxin and Ethylene Signaling Pathways Is Involved in *Arabidopsis* Growth Promotion by *Burkholderia phytofirmans* PsJN

**DOI:** 10.3389/fpls.2016.00492

**Published:** 2016-04-12

**Authors:** María J. Poupin, Macarena Greve, Vicente Carmona, Ignacio Pinedo

**Affiliations:** ^1^Laboratorio de Bioingeniería, Facultad de Ingeniería y Ciencias, Universidad Adolfo IbáñezSantiago, Chile; ^2^Center for Applied Ecology and SustainabilitySantiago, Chile; ^3^Millennium Nucleus Center for Plant Systems and Synthetic BiologySantiago, Chile

**Keywords:** rhizobacteria, EIN2, IAA1, NPA, AVG, ETO2, root development, root hairs

## Abstract

Modulation of phytohormones homeostasis is one of the proposed mechanisms to explain plant growth promotion induced by beneficial rhizobacteria (PGPR). However, there is still limited knowledge about the molecular signals and pathways underlying these beneficial interactions. Even less is known concerning the interplay between phytohormones in plants inoculated with PGPR. Auxin and ethylene are crucial hormones in the control of plant growth and development, and recent studies report an important and complex crosstalk between them in the regulation of different plant developmental processes. The objective of this work was to study the role of both hormones in the growth promotion of *Arabidopsis thaliana* plants induced by the well-known PGPR *Burkholderia phytofirmans* PsJN. For this, the spatiotemporal expression patterns of several genes related to auxin biosynthesis, perception and response and ethylene biosynthesis were studied, finding that most of these genes showed specific transcriptional regulations after inoculation in roots and shoots. PsJN-growth promotion was not observed in *Arabidopsis* mutants with an impaired ethylene (*ein2-1*) or auxin (*axr1–5*) signaling. Even, PsJN did not promote growth in an ethylene overproducer (*eto2*), indicating that a fine regulation of both hormones signaling and homeostasis is necessary to induce growth of the aerial and root tissues. Auxin polar transport is also involved in growth promotion, since PsJN did not promote primary root growth in the *pin2* mutant or under chemical inhibition of transport in wild type plants. Finally, a key role for ethylene biosynthesis was found in the PsJN-mediated increase in root hair number. These results not only give new insights of PGPR regulation of plant growth but also are also useful to understand key aspects of *Arabidopsis* growth control.

## Introduction

Plants live in close association with microorganisms that are fundamental for their performance and survival. Among these microorganisms, plant growth-promoting rhizobacteria (PGPR) induce positive effects in plants, such as increased growth or reduced susceptibility to biotic and abiotic stresses (Lugtenberg and Kamilova, 2009; Yang et al., 2009; Berg et al., 2014; Liu and Zhang, 2015). The growing demand for alternatives to the massive use of agrochemicals has increased interest to exploit and understand the advantages of using these microorganisms for a more environmentally sustainable agricultural management (Mitter et al., 2013). However, the genetic and molecular mechanisms governing plant responses to PGPR have only recently started to be understood (Verhagen et al., 2004; Wang et al., 2005; Lopez-Bucio et al., 2007; Cartieaux et al., 2008; Contesto et al., 2010; Schwachtje et al., 2012; Poupin et al., 2013; Zamioudis et al., 2013). One of the proposed mechanisms to explain growth promotion induced by PGPR is phytostimulation, either through the microbial production of phytohormones, or by modulation of their homeostasis in plants (Lugtenberg and Kamilova, 2009). In this regard, the effect of different bacterial strains producing phytohormones has been reported. For example, about 80% of bacteria from the rhizosphere are able to produce indole acetic acid (IAA, the predominant naturally occurring auxin in plants), indicating a possible role in interaction with the plant (Ahmed and Hasnain, 2014; Duca et al., 2014). Plant-exuded tryptophan would enter the IAA biosynthesis pathways of the bacteria living in the rhizosphere, and a significant part of the bacterial IAA would be delivered to the plant root (Spaepen et al., 2007). For instance, Spaepen et al. (2014) reported that bacterial auxin biosynthesis by *Azospirillum brasilense* is important to reduce primary root length in *Arabidopsis*. Interestingly, Zuniga et al. (2013) reported that the *Arabidopsis* primary root growth induced by *Burkholderia phytofirmans* PsJN was associated to the bacterial auxin degradation capacity. Other reports have described the role of gibberellin-producing rhizobacteria in plant growth promotion (Bottini et al., 2004; Joo et al., 2005; reviewed in Boiero et al., 2007; Kang et al., 2012, 2014; Khan et al., 2014). Less is known about the responses of hormonal plant-signaling pathways *in planta* upon interaction with PGPR. Contesto et al. (2010) reported that *Phyllobacterium brassicacearum* STM196 increased *Arabidopsis* lateral roots triggering changes in IAA distribution and homeostasis independently from bacterial IAA. A role for auxin signaling and transport was also proposed for *Pseudomonas* strains in *Arabidopsis* (Zamioudis et al., 2013). In contrast, Lopez-Bucio et al. (2007) reported that *Bacillus megaterium* promotes growth in an auxin and ethylene independent mechanism. Similarly, the importance of other phytohormones such as citokinins (Ortiz-Castro et al., 2008; Cassán et al., 2009; Liu et al., 2013) has been reported in these plant-bacteria interactions. Moreover, *Herbaspirillum frisingense* GSF30 induced long-term differences in genes associated to jasmonate and ethylene signaling in the perennial energy grass Miscanthus (Straub et al., 2013). Besides this important information, the molecular interplay among plant hormones that lead to plant growth promotion under PGPR inoculation is not well understood. Phytohormones regulate almost every aspect of plant growth and development. But far from acting in linear pathways, over recent years it has become clear that a complex network of interactions and feedback circuits among hormonal pathways determines the developmental outcome (Růzicka et al., 2007; Kazan, 2013; Bar and Ori, 2014). In this context, crosstalk mechanisms between auxin and ethylene have been well defined in the regulation of different aspects of plant architecture such as, hypocotyl elongation (Smalle et al., 1997), apical hook development (Vandenbussche et al., 2010), root hair growth and differentiation (Pitts et al., 1998), root gravitropism (Lee et al., 1990), root growth (Pickett et al., 1990; Rahman et al., 2001; Růzicka et al., 2007; Lewis et al., 2011) and lateral root initiation and emergence (Ivanchenko et al., 2008).

*Burkholderia phytofirmans* PsJN is one of the best-studied PGPR, which inhabit the rhizosphere and endosphere, promoting growth and enhancing stress adaptation in several herbaceous and woody plant species (Compant et al., 2005; Sessitsch et al., 2005; Ait Barka et al., 2006; Bordiec et al., 2011; Da et al., 2012; Fernandez et al., 2012; Kim et al., 2012; Naveed et al., 2014). This strain is also capable of promoting growth and accelerating the whole life cycle of *Arabidopsis thaliana* plants (Poupin et al., 2013; Zuniga et al., 2013). In *Arabidopsis*, strain PsJN induces primary root growth, root hair development/growth, promotes aerial growth increasing the epidermal cell size (Poupin et al., 2013) and induces salt-stress tolerance (Pinedo et al., 2015). Also, a transcriptome analysis showed that inoculation of *A. thaliana* with *B. phytofirmans* PsJN regulated the expression of several genes related to the auxin signaling pathway in plants (Poupin et al., 2013).

To assess whether the plant architecture modifications induced by PGPR may be explained by changes in the plant’s ethylene and auxin signaling, we used *A. thaliana* plants and the PGPR *B. phytofirmans* PsJN as a study model. Then, the spatiotemporal expression patterns of *A. thaliana* genes, involved in the synthesis, perception, transport, or signaling pathway of these phytohormones were analyzed in inoculated plants. Mutant seeds impaired in auxin/ethylene signaling and a chemical approach to inhibit auxin polar transport and/or ethylene synthesis in plants, were also used to confirm the role of both hormones in the plant growth induced by strain PsJN. Thus, this work discusses the influence of these plant’s hormones, discriminating their role in different aspects of the growth promotion induced by this PGPR strain.

## Materials and Methods

### Plant Growth Conditions and Treatments

*Burkholderia phytofirmans* PsJN was routinely grown in minimal saline Dorn medium (Dorn et al., 1974), containing 10 mM fructose, in an orbital shaker (150 rpm) at 30°C. Cell suspensions from each inoculum were then collected and adjusted to approximately 10^8^ colony forming units per milliliter (CFU/ml), as determined by plate counting. Seeds of *A. thaliana* were surface sterilized with 50% sodium hypochlorite (100% commercial laundry bleach) containing 0.1% Tween 20, rinsed three times with sterile water, and kept at 4°C for 4 days to synchronize germination. Then, seeds were sown on square Petri dishes with half strength Murashige and Skoog medium 0.8% agar (MS; Murashige and Skoog, 1962), and 10^4^ CFU/ml of bacteria. Plates were placed vertically in a growth chamber at 22°C with a photoperiod of 16 h of light and 8 h of dark. At day 14 after sowing (14 DAS) different growth parameters were determined in plants. Seeds of *Arabidopsis* Col-0, *eir1-1* (CS8058), *ein2-1* (CS3071), *axr5-1* (CS16234), and *eto2* (CS8059) were obtained from ABRC. 1-*N*-naphthylphthalamic acid (NPA; Fluka Sigma–Aldrich) was prepared in DMSO in a 50 mM stock and used at a final concentration of 2 μM diluted in MS. Amino ethoxyvinyl glycine hydrochloride (AVG; Fluka Sigma–Aldrich) was prepared in a stock with sterile water at 10 mM and used at a final concentration of 1.5 uM.

### Phenotypical Analysis and Statistics

Fresh weight of plants was determined with a Shimadzu analytical balance (Shimadzu Corporation, Japan). Growth of primary roots was registered using a standard cm/mm ruler. The number and length of root hairs was analyzed at the root tip (5 mm) with a stereo microscope (Leica S6D, Germany). Images of root tips and rosette areas were also calculated using the ImageJ software.

To test for significant differences in response variables, one or two-way ANOVA were performed, using Kolmogorov–Smirnov and D’Agostino & Pearson tests for normality, and Hartley and Bartlett tests for homogeneity of variances (*P* < 0.05). Statistical analyses were carried out using the General Linear Models option in the statistical software Prism Graphpad 5 (GraphPad Software, Inc., La Jolla, CA, USA). When differences in the means were significant, a Tukey’s HSD test was performed (Sokal and Rohlf, 1969).

### RNA Extraction, cDNA Synthesis, and qRT-PCR Analyses

Seeds were sown as described above without PsJN inoculation. Over 7 DAS, plantlets were transferred to a fresh medium containing or not PsJN strain. Roots and aerial zones from 5 to 13 plants per replica were collected at 2, 24, 48, and 96 h after treatments. For RNA extraction, samples were grinded with a pestle in an Eppendorf tube. Then, RNA was obtained using the Trizol^®^(Invitrogen^TM^, USA) method following the manufacturer’s instructions. For cDNA synthesis, 1 μg of total RNA treated with DNAse I (RQ1, Promega, USA) was reverse transcribed with random hexamer primers using the Improm II reverse transcriptase (Promega, USA), according to the manufacturer’s instructions. Real time RT-PCR was performed using the Brilliant^®^SYBR^®^Green QPCR Master Reagent Kit (Agilent Technologies, USA) and the Eco Real-Time PCR detection system (Illumina^®^, USA) as described by Poupin et al. (2013). The PCR mixture (15 μl) contained 2.0 μl of template cDNA (diluted 1:10) and 140 nM of each primer. Amplification was performed under the following conditions: 95°C for 10 min, followed by 40 cycles of 94°C, 30 s; 57–65°C, 30 s; and 72°C, 30 s, followed by a melting cycle from 55 to 95°C. Relative gene expression calculations were conducted as described in the software manufacturer’s instructions: an accurate ratio between the expression of the gene of interest (GOI) and the housekeeping (HK) gene was calculated according to equation: 2^-(ΔCtGOI-HK)^. Then, gene expression levels were normalized to the average value of the treatment with less expression. Expression of three housekeeping genes was analyzed for treatments *AtSAND* (AT2G28390), *PP2A* (AT1G13320), and *TIP41-like* (AT4G34270), using described PCR primer pairs (Czechowski et al., 2005). In all cases, expression of HK genes was highly stable and similar results were obtained using them as normalization genes. Data presented here represent the normalization using *AtSAND* amplification. Primer pairs designed in this study were obtained using Primer Express v.2.0 (Applied Biosystems, USA) and confirmed with Primer-BLAST (NCBI). Sequences of all primers and their references (if applicable) are listed in **Supplementary Table S1**. In all cases the reaction specificities were tested with melt gradient dissociation curves and electrophoresis gels (agarose 2%) of each PCR product. All experiments were performed in three to five biological, and two technical, replicates.

## Results

### Several Plant-Genes Related to the Auxin Pathway are Regulated by *B. phytofirmans* PsJN

As a first approach to analyze the role of auxin associated pathways in the *Arabidopsis*-growth promotion induced by strain PsJN, the transcriptional profiles of a selected group of genes involved in the synthesis, perception, transport, or signaling of this hormone were analyzed in *A. thaliana* roots and shoots. Thus, sterile *A. thaliana* Col-0 seed were sown on MS sterile medium. Seven days after sowing (DAS), plantlets were transplanted to the same culture media with or without a PsJN inoculum. Then, roots and aerial tissues of plants were collected after 2, 24, 48, and 96 h of treatment and then processed to extract RNA and to obtain cDNA. Afterward, gene expression patterns were analyzed by real time RT-PCR as described in Poupin et al., 2013. Statistical significant differences were calculated using one-way ANOVA comparing the inoculation effects at each measured time.

**FIGURE 1:**
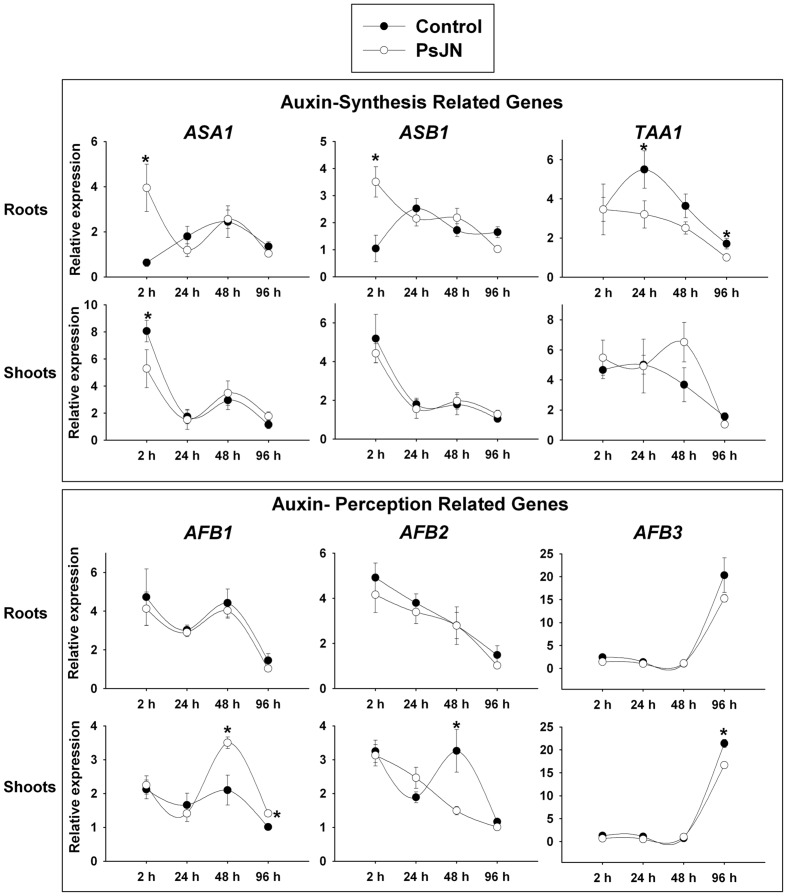
**Temporal quantitative real-time PCR of selected *Arabidopsis* genes involved in auxin synthesis and perception after inoculation with *Burkholderia phytofirmans* PsJN.** Relative gene expression of selected genes involved auxin synthesis and perception in roots and shoots at 2, 24, 48, and 96 h post inoculation (hpi) with the strain. Data are means ± SE of 3–5 biological replicates per treatment, each considering tissue from 5 to 13 plants and two technical replicates. Normalization was performed with the housekeeping *SAND* family gene (AT2G28390). Asterisk indicates statistical significance among treatments in a particular time (One way ANOVA, *p* < 0.05).

The expression patterns of genes involved in auxin synthesis were studied first. The *ASA1* (*ANTHRANILATE SYNTHASE ALPHA SUBUNIT 1*) and the *ASB1* (*ANTHRANILATE SYNTHASE BETA SUBUNIT 1*) genes catalyze the rate-limiting step and the first step of tryptophan (Trp) biosynthesis, which is a precursor of auxin biosynthesis (Mano and Nemoto, 2012). Here, *ASA1* and *ASB1* were significantly up-regulated in roots 2 h post inoculation (hpi) and *ASA1* was down-regulated in shoots at the same time after inoculation (**Figure 1**). TAA1 (TRYPTOPHAN AMINOTRANSFERASE OF *ARABIDOPSIS* 1) is a Trp aminotransferase related protein, which catalyzes the key step of auxin biosynthesis through the conversion of Trp to indole-3-pyruvic acid, thus positively directing auxin biosynthesis (Stepanova et al., 2008; He et al., 2011). *TAA1* was down-regulated by inoculation at 24 and 96 hpi in roots, and was not significantly affected in shoots (**Figure 1**).

**FIGURE 2:**
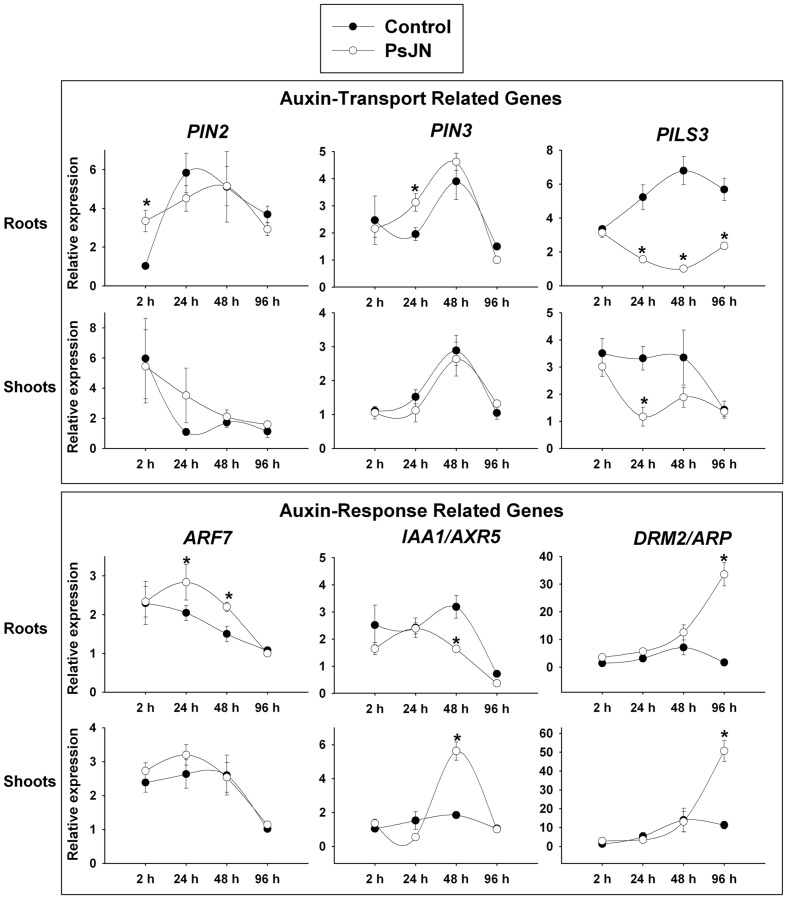
**Temporal quantitative real-time PCR of selected *Arabidopsis* genes involved in auxin transport and response after inoculation with *B. phytofirmans* PsJN.** Relative gene expression of selected genes involved auxin transport and response in roots and shoots at 2, 24, 48, and 96 hpi with the strain. Data are means ± SE of 3–5 biological replicates per treatment, each considering tissue from 5 to 13 plants and two technical replicates. Normalization was performed with the housekeeping *SAND* family gene (AT2G28390). Asterisk indicates statistical significance among treatments in a particular time (One way ANOVA, *p* < 0.05).

Auxin is perceived by a transient co-receptor complex consisting of TIR1 (TRANSPORT INHIBITOR RESPONSE1) and AFB (AUXIN SIGNALING F-BOX1–3) proteins (Salehin et al., 2015). While *AFB1–3* were not regulated by inoculation in roots at any of the evaluated times, in shoots *AFB1* was up-regulated at 48 and 96 hpi; *AFB2* was down-regulated by the treatment at 48 hpi and *AFB3* was down-regulated at 96 hpi (**Figure 1**). Concerning auxin transport, the gene expression patterns of *PIN2*, *PIN3*, and *PILS3* were analyzed under inoculation (**Figure 2**). *PIN2* (*PIN FORMED 2*), also known as *EIR1* (*ETHYLENE INSENSITIVE ROOT 1*) codes for an efflux polar transporter (Luschnig et al., 1998) and showed an up-regulation 2 hpi in roots. PIN3 (PIN FORMED 3) is also involved in auxin efflux and is expressed in gravity-sensing tissues, with PIN3 protein accumulating predominantly at the lateral cell surface (Friml et al., 2002). This last gene was up-regulated in roots 24 hpi (**Figure 2**). Neither *PIN2* nor *PIN3* showed a regulation by inoculation in shoots, consistently with their predominant expression in roots. *PIN1* and *PIN7* were also studied, showing no regulation after inoculation (data not shown). Using a transcriptomic analysis we previously found that *PILS3* (*AT1G76520*), a gene putatively involved in auxin transport (Barbez et al., 2012), is significantly down-regulated in whole plants by strain PsJN (Poupin et al., 2013). This gene belongs to a family of novel putative auxin transporters called PIN-LIKES (PILS). These proteins are required for auxin-dependent regulation of plant growth by determining the cellular sensitivity to auxin. PILS proteins regulate intracellular auxin accumulation at the endoplasmic reticulum and thus auxin availability for nuclear signaling. PILS activity affects the level of endogenous IAA, presumably via intracellular accumulation and metabolism (Barbez et al., 2012). Here, *PILS3* was down-regulated in shoots at 24 hpi and severely down-regulated in roots from 24 hpi (**Figure 2**).

Also, the expression pattern of the genes involved in auxin response *ARF7*, *IAA1*, and DRM2/ARP were analyzed. ARF7 is an AUXIN RESPONSE FACTOR, which is a transcriptional activator of early auxin responsive genes, and is involved in lateral root formation (Okushima et al., 2007). This gene was up-regulated in roots 24 and 48 hpi and was not affected in shoots.

Aux/IAA proteins are short-lived transcriptional factors that function as repressors of early auxin response genes at low auxin concentrations. Repression is thought to result from the interaction with auxin response factors (ARFs), which are proteins that bind to the auxin-responsive promoter element (AuxRE). Formation of heterodimers with ARF proteins may alter their ability to modulate early auxin-response genes expression (Liscum and Reed, 2002). *IAA1* (*INDOLE-3-ACETIC ACID INDUCIBLE 1*), also known as *AXR5* (*AUXIN RESISTANT 5*) is a member of the AUX/IAA family. *IAA1* gene is expressed throughout development with the highest level of RNA accumulation in roots, inflorescences, and flowers. In addition, the gene is induced rapidly by auxin and expression remains high after prolonged exposure to auxin (Yang et al., 2004). This gene presented a down-regulation in inoculated roots 48 hpi and an up-regulation at the same time in shoots (**Figure 2**). Finally, the expression pattern of (DRM2/ARP, DORMANCY ASSOCIATED GENE-1/AUXIN-REPRESSED PROTEIN; *At2g33830*) was studied after inoculation. DRM1 and 2 are proteins related to biotic or abiotic stress response (Rae et al., 2014). *DRM2* presented a high up-regulation (more than 30 times) 96 hpi in both tissues (**Figure 2**).

### *Burkholderia phytofirmans* PsJN Regulates Ethylene-Biosynthesis Related Genes

**FIGURE 3:**
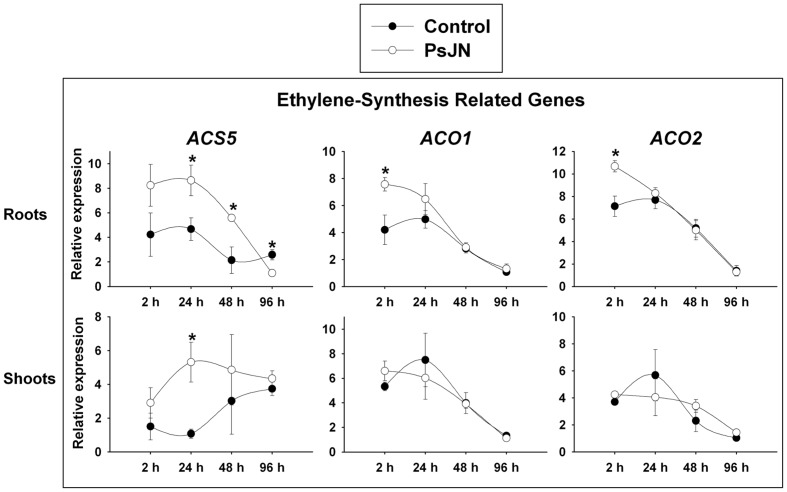
**Temporal quantitative real-time PCR of selected *Arabidopsis* genes related to ethylene biosynthesis after inoculation with *B. phytofirmans* PsJN.** Relative gene expression of selected genes involved in the ethylene biosynthesis pathway in roots and shoots at 2, 24, 48, and 96 hpi with the strain. Data are means ± SE of 3–5 biological replicates per treatment, each considering tissue from 5 to 13 plants and two technical replicates. Normalization was performed with the housekeeping SAND family gene (AT2G28390). Asterisk indicates statistical significance among treatments in a particular time (One way ANOVA, *p* < 0.05).

Ethylene gas is a crucial regulator of numerous aspects of plant development and physiology, including germination; seedling growth and morphology; fruit ripening; organ senescence; and stress/defense responses (Merchante et al., 2013; Broekgaarden et al., 2015). Ethylene biosynthesis is generally maintained at low levels and rapidly increases under external or internal signals and its regulation depends on transcriptional and post translational mechanisms that regulate the activity levels of the biosynthetic enzymes (Booker and DeLong, 2015). In *Arabidopsis*, ethylene is produced from methionine through the conversion of *S*-adenosyl-L-methionine in 1-aminocyclopro-pane-1-carboxylic acid (ACC) by ACC synthase (ACS), and then ACC is converted in ethylene by ACC oxidase (ACO) (Kende, 1993). ACS and ACO are encoded by multigene families and regulated by many biotic and abiotic factors (Babula et al., 2006; Datta et al., 2015). To assess a role for the ethylene pathway in the growth promotion induced by strain PsJN, the expression patterns of one member of the ACS family and two of the ACO were analyzed after inoculation (**Figure 3**). The *Arabidopsis* genome encodes nine ACS polypeptides that form eight functional homodimers (Tsuchisaka and Theologis, 2004a). *ACS5*, which is one of the genes that is highly expressed in roots and responsive to auxin (Tsuchisaka and Theologis, 2004b), was up-regulated in inoculated roots at 24 and 48 hpi, and down-regulated at 96 hpi (**Figure 3**). In shoots of inoculated plants the gene also presented an up-regulation 24 hpi (**Figure 3**). Moreover, *ACO1* and *ACO2* were up-regulated only in roots 2 hpi (**Figure 3**). The expression patterns of the genes associated to ethylene signaling, *EIN2* and *CTR* were not significantly regulated by the inoculation (data not-shown).

### Phytostimulation Induced by Strain PsJN is Affected by Auxin Transport and/or Ethylene Biosynthesis Inhibition

**FIGURE 4:**
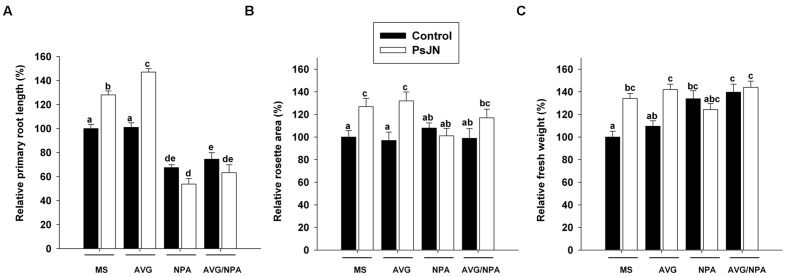
**Short-term effects of auxin transport inhibition and/or ethylene biosynthesis inhibition in *Arabidopsis* growth induction mediated by *B. phytofirmans* PsJN.** Relative primary root length **(A)**, rosette area **(B)**, and fresh weight **(C)** in 14 days old plants inoculated (PsJN) or not (control) with strain PsJN. Plants were also grown without chemical inhibitors (MS), or under the chemical treatment with the auxin efflux inhibitor NPA (*N*-1-naphthylphthalamic acid) at 2 μM and/or with the ethylene biosynthesis inhibitor amino ethoxyvinyl glycine hydrochloride (AVG) at 1.5 μM. Data are means ± SE of at least 15 plants per treatment. Lowercase letters on top of the bars (a–e) indicate significant statistical differences among treatments. Bars that do not share an identical letter have significant differences, according to a Two-way ANOVA analysis (*p* < 0.05), considering inoculation and chemical treatments as factors, and a Tukey *post hoc* test (*p* < 0.05). Results are representative of three different experiments.

*Burkholderia phytofirmans* PsJN treatment produced a significant increase in the primary root length (**Figure 4A**), rosette area (**Figure 4B**) and fresh weight of *Arabidopsis* plants (**Figure 4C**). To get a better understanding of auxin role in the growth-promoting effects of strain PsJN, the inhibitor of auxin polar transport NPA (1-*N*-naphthylphthalamic acid, Murphy et al., 2000) was used with the inoculation treatments. This compound inhibits primary root growth, primarily by reducing cell production rate, but does not affect the localization of auxin efflux proteins (PIN1 or PIN2) (Rahman et al., 2007). When NPA was added to the medium, the primary roots were significantly smaller (67.4 ± 2.3%; **Figure 4A**) than those of the control treatment. Strain PsJN did not induce primary root growth in the presence of this inhibitor (53.7 ± 4.7%; **Figure 4A**). NPA treatment did not affect the rosette area, but PsJN inoculation did not increase this parameter when plants were inoculated and treated with NPA (**Figure 4B**). The treatment with NPA significantly increased fresh weight in plants (133.9 ± 7.0%) but a synergistic/additive effect was not observed when plants were inoculated and treated with NPA at the same time (**Figure 4C**).

The ethylene synthesis inhibitor AVG hydrochloride (Sun et al., 2010) was used to further investigate a possible mediation of this hormone in *Arabidopsis*-growth induced by strain PsJN. At the used concentrations AVG did not affect the measured growth parameters and strain PsJN significantly increased these parameters in the presence of the inhibitor (**Figures 4A–C**). When both inhibitors were incorporated at the same time, strain PsJN did not affected primary roots and fresh weight similarly to the NPA treatment (**Figures 4A,C**) and a slight but not significant increase in rosette area was observed (**Figure 4B**).

**FIGURE 5:**
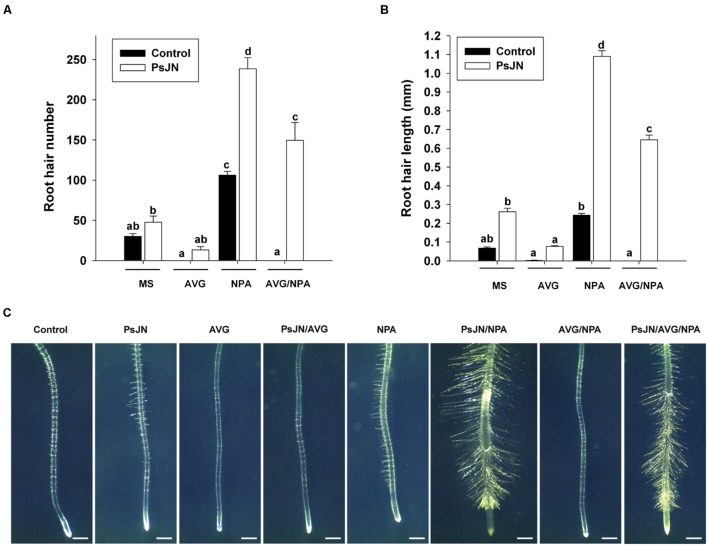
**Effects of *B. phytofirmans* PsJN in root hair number and length under auxin transport and/or ethylene biosynthesis inhibition.** Graphic representation of the root hair number **(A)** and hair length **(B)** of root tips (5 mm) from plants inoculated (PsJN) or not (control) with *B. phytofirmans* PsJN. Plants were also grown without chemical inhibitors (MS), or under the treatment with the auxin efflux inhibitor NPA (*N*-1-naphthylphthalamic acid) at 2 μM and/or with the ethylene biosynthesis inhibitor amino ethoxyvinyl glycine hydrochloride (AVG; Fluka Sigma–Aldrich) at 1.5 μM. Data are means ± SE of at least 20 plants per treatment. Lowercase letters on top of the bars (a–d) indicate significant statistical differences among treatments. Bars that do not share an identical letter have significant differences, according to a Two-way ANOVA analysis (*p* < 0.05), considering inoculation and chemical treatments as factors, and a Tukey *post hoc* test (*p* < 0.05). Results are representative of three different experiments. Representative photographs of each treatment are presented in **(C)**; white bars in photographs correspond to 1 mm.

Also, the effect of *B. phytofirmans* PsJN and these inhibitors was analyzed in the number and length of *Arabidopsis* root hairs (**Figure 5**). Strain PsJN significantly increased the number (**Figures 5A,C**) and length (**Figures 5B,C**) of root hairs at the root tip. On the contrary, root hairs were almost not detected under AVG treatment, and this effect was not significantly restored by PsJN inoculation (**Figures 5A–C**). NPA treatment significantly increased both parameters (**Figures 5A–C**). Interestingly the root hair length and number were highly augmented when plants were inoculated with strain PsJN and treated with NPA concomitantly (**Figures 5A–C**). Likewise with AVG treatment, when NPA was added together with AVG the root hairs were not detected, but this effect was counteracted when plants were also inoculated (**Figures 5A–C**).

### *Burkholderia phytofirmans* PsJN Does Not Induce Growth in Mutants Impaired in Auxin or Ethylene Pathways

**FIGURE 6:**
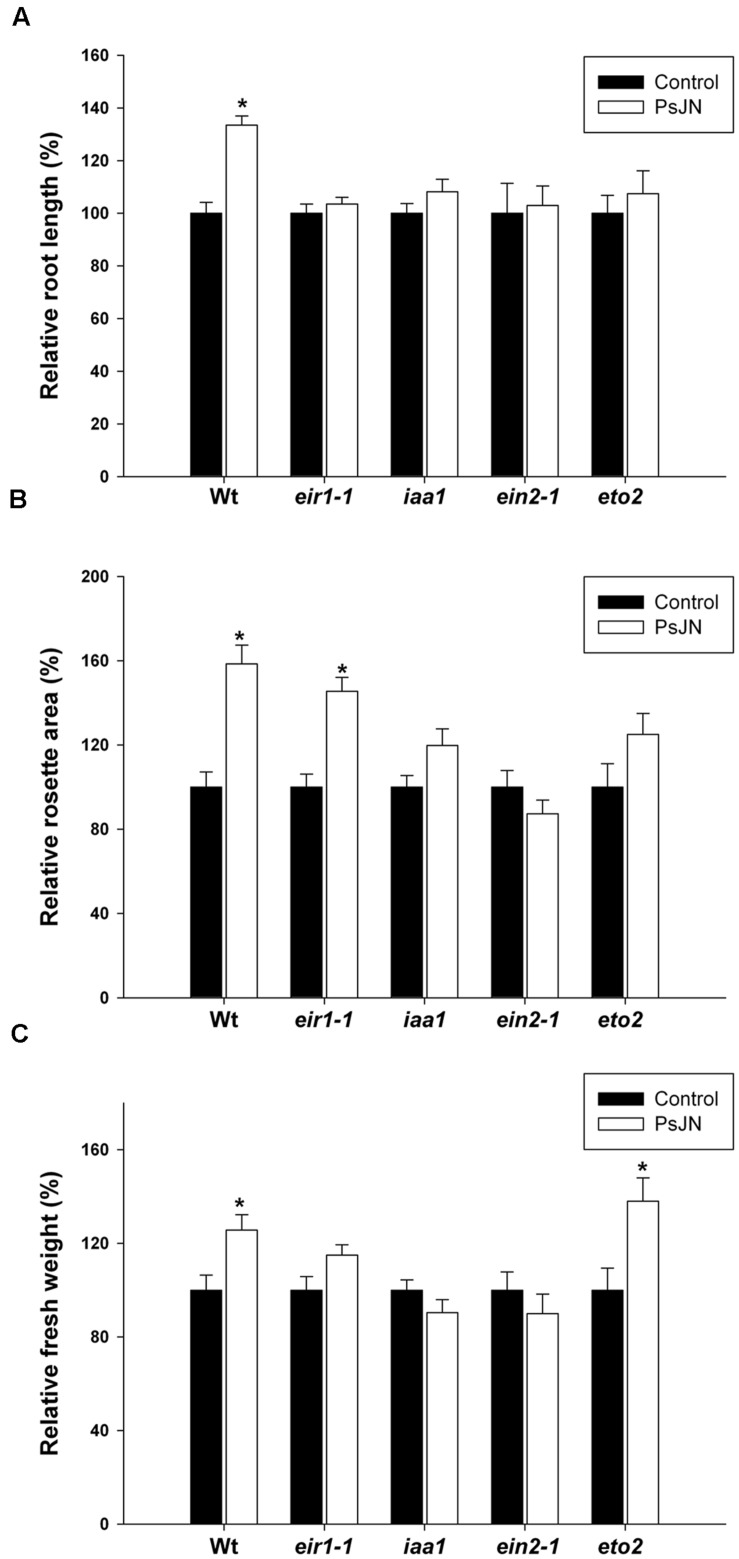
**Effects of *B. phytofirmans* PsJN in different *Arabidopsis* mutants associated to auxin and ethylene pathways.** Relative primary root length **(A)**, rosette area **(B)** and fresh weight **(C)** in 14 days old wild type (wt) or mutant plants inoculated (PsJN) or not (control) with strain PsJN. *Arabidopsis* mutants lines used were *eir1–1* (CS8058), *iaa1* (CS16234), *ein2-1* (CS3071), and *eto2* (CS8059). Data are means ± SE of 17–35 plants per treatment, and represent at least four independent experiments. Asterisk indicates statistical significance between the inoculated and non-inoculated plants in each genotype (One way ANOVA, *p* < 0.05).

In addition, the effects of strain PsJN in different *A. thaliana* mutant lines associated to auxin or ethylene pathway were tested (**Figure 6**). *eir1–1*, a mutant in *EIR1/PIN2* (ethylene insensitive root 1; Roman et al., 1995; Luschnig et al., 1998) was inoculated to confirm the role of auxin polar transport in growth-promotion induced by strain PsJN. *EIR1* is part of a plant gene family with similarities to bacterial membrane transporters and encodes an auxin efflux carrier. This mutant has agravitropic roots and has reduced root sensitivity to ethylene (Roman et al., 1995; Luschnig et al., 1998; Stepanova et al., 2007). It also fails to respond to internally generated auxin, but reacts normally to externally applied auxin (Luschnig et al., 1998). In the *eir1–1* mutant, strain PsJN did not promote root growth or relative fresh weight (**Figures 6A,C**) but significantly increased the rosette area (**Figure 6B**).

Auxin signaling is mediated by at least three sets of proteins: the ARFs, the Aux/IAAs, and the F-box protein TIR1 (Salehin et al., 2015). The ARF proteins bind DNA and directly activate or repress transcription of target genes, the Aux/IAA proteins repress ARF function and TIR1 promotes the degradation of the Aux/IAA transcriptional repressors (Calderon Villalobos et al., 2012). *IAA1/AXR5* codes for the protein IAA1, and the *iaa1/axr5* mutant plants are resistant to auxin and display a variety of auxin-related growth defects including defects in root and shoot tropisms (Yang et al., 2004). In the presence of auxin, TIR1 interacts with AXR5/IAA1 mediating its degradation in the proteasome, the *axr5-1* mutation results in an amino acid substitution that prevents the interaction with TIR1, inducing the accumulation of IAA1/AXR5 in presence of auxin (Yang et al., 2004). Further, the *axr5-1* mutation results in a decrease in auxin-regulated transcription. AXR5 is expressed throughout plant development consistent with the pleiotropic mutant phenotype. Remarkably, none of the growth parameters were significantly increased when *iaa1/axr5-1* mutants were inoculated with strain PsJN (**Figures 6A–C**).

Also the mutant plants *ein2-1* and *eto2* were analyzed to confirm the role of ethylene in the PsJN-plant growth promotion. EIN2 (ETHYLENE INSENSITIVE 2) acts as a positive regulator of the ethylene pathway, activating transcription factors of the EIN3 family located in the nucleus. EIN3 binds to promoters of ETHYLENE RESPONSE FACTORS (ERFs) genes and stimulate their transcription in an ethylene-dependent manner. These ERF regulate the expression of many downstream genes (Stepanova and Alonso, 2005). Alike *iaa1/axr5-1*, PsJN was not able to induce growth in a mutant of *EIN2* (*ein2-1*) (**Figures 6A–C**). *ETHYLENE OVERPRODUCER 2* (*ETO2*) codes for ACC SYNTHASE 5 (ACS5), and *eto2* is a dominant mutant which overproduces ethylene increasing the half-life of the protein (Chae et al., 2003). The inoculation with strain significantly increased fresh weight (**Figure 6C**) in this mutant and did not increase the other two parameters (**Figures 6A,B**). Additionally, the number and length of root hairs were measured in the *eir1–1* and *eto2* mutants, finding that inoculation with strain PsJN in both mutants did not modify the first parameter but increased the root hair length (**Supplementary Figure S1**).

## Discussion

Auxin plays a crucial role in the regulation of almost every plant development aspect, from embryo formation to organ senescence (Brumos et al., 2014). The responses to this hormone depend on the context and can involve changes in cell division, cell expansion, and cell fate. Recently, it was found that the auxin-responsive set of genes differs significantly between different cell types in the root, reflecting complex response mechanisms to this hormone in plants (Salehin et al., 2015). Ethylene is also fundamental for several growth processes and environmental responses in plants and crosstalk mechanisms between both hormones have been defined in the regulation of different aspects of plant development (Pitts et al., 1998; Růzicka et al., 2007; Lewis et al., 2011; Vandenbussche et al., 2012). Although ethylene and auxin signaling pathways are relatively well characterized, the mechanisms of their interaction are still unclear (Růzicka et al., 2007). These hormones may have agonistic or antagonistic effects on different plant growth processes, which seems to be linked to a very precise localization and homeostasis of both hormones in different cells and plant tissues (Muday et al., 2012). Some of the agonistic/synergistic roles of these hormones are the inhibition of primary root elongation and the promotion of root hair formation (Růzicka et al., 2007), while in processes such as lateral root formation and hypocotyl elongation; they act antagonistically (Lewis et al., 2011). Ethylene influences many features of auxin-dependent seedling growth by altering auxin signaling, synthesis or transport, or in many cases all three (Jung and McCouch, 2013). The interactions are even more complex for processes in which auxin is accumulated asymmetrically to drive differential growth, such as gravitropism, or hook opening, where ethylene prevents this asymmetry by either lowering or raising auxin accumulation on both sides of these organs (Muday et al., 2012). Different concentration of these hormones can induce different outcomes. For instance, an enhanced ethylene biosynthesis, resulting from the application of low doses of ACC, promotes the initiation of lateral root primordia, but treatments with higher doses inhibit the ability of pericycle to initiate lateral root primordia, favoring the growth of already existing lateral roots (Ivanchenko et al., 2008; Negi et al., 2008).

Interesting studies have reported the role of particular plant signaling hormones such as auxin in plant responses to PGPR (Zhang et al., 2007) and especially describing their participation in controlling root development (Contesto et al., 2010; Zamioudis et al., 2013; Spaepen et al., 2014). Nevertheless, the interplay of different hormonal pathways explaining the beneficial effects of these bacteria in plants is far from being understood. Considering the increasing evidence of auxin and ethylene crosstalk controlling plant development, here the role of both hormonal signaling pathways was analyzed in the context of growth promotion of *A. thaliana* plants inoculated with *B. phytofirmans* PsJN. This was approached analyzing the spatiotemporal expression patterns of several genes associated to both hormonal pathways and studying the effects of strain PsJN under chemical disruption of auxin transport and/or ethylene biosynthesis and finally using mutant plants with impaired auxin/ethylene signaling or transport.

### Role of the Auxin Signaling Pathway in *Arabidopsis* Growth Promotion Induced by Strain PsJN

Auxin is transported from cell to cell and in shoots IAA moves unidirectionally from the apex to the base (Zazimalova et al., 2010). In roots, auxin is transported in two directions: through the central cylinder toward the root tip (Rootward) and through the outer layers of root cells in the shootward direction (Baskin et al., 2010). In *Arabidopsis* roots, both polarities of IAA movement control distinct processes and are mediated by unique sets of auxin transport proteins (Muday et al., 2012). Rootward movement of IAA from the shoot into the root apex has been implicated in the control of lateral root formation and elongation, involving influx transport proteins such as AUX1 and efflux auxin proteins such as PIN1, PIN3, and PIN7 (Lewis et al., 2011). Shootward movement is required for gravitropism and PIN2 (an efflux transporter), among others, mediates this response (Rashotte et al., 2001; Rahman et al., 2010). *B. phytofirmans* PsJN induced the expression of the auxin efflux root transporters genes *PIN2* and *PIN3* (**Figure 2**). Consistently, strain PsJN did not induce growth in the primary root, neither in the *eir1-1/pin2* mutant (**Figure 6**) nor under chemical auxin efflux transport inhibition (NPA, **Figure 4**). These results indicate that auxin efflux is fundamental for the PsJN-modulation of *Arabidopsis* root growth. The root is dependent on several sources of IAA in both, proximal and distal. However, a substantial part of the *de novo* synthesized IAA observed in the root is synthesized in the aerial parts of the plant and transported down to the root system. NPA treatment blocks auxin polar-transport, trapping IAA in source tissues such as developing leaves (Ljung et al., 2005). Non-inoculated NPA treated plants presented increased fresh weight and PsJN did not induce a synergistic effect in this parameter under NPA treatment (**Figure 4**). This suggests that fresh weight augmentation, induced by strain PsJN, converges into the same molecular signaling pathway affected by NPA treatment and depending on auxin accumulation/production in the aerial tissues. Additionally, the expression of *PILS3* presumably involved in intracellular accumulation and metabolism of IAA (Barbez et al., 2012), was down-regulated in roots by PsJN inoculation (**Figure 2**). Thus, strain PsJN may regulate IAA compartmentalization not only in different root tissues/layers but also in the intracellular compartments. Further and complex analysis of the specific and *in situ* localization and accumulation of auxin might be helpful to prove this hypothesis.

Considering auxin biosynthesis, *ASA1* and *ASA2* (involved in tryptophan biosynthesis) were transiently activated in inoculated roots. TAA1 mediates local auxin biosynthesis and inhibition of root growth (Stepanova et al., 2008; Yang et al., 2014). Ethylene signaling regulates *TAA1*, thus positively directing auxin biosynthesis (Stepanova et al., 2008; He et al., 2011). Interestingly, this gene was down-regulated in roots by strain PsJN in accordance with the primary root growth promotion induced by this strain.

Auxin perception pathway was also affected under PsJN inoculation, the three AFB genes presented a regulation only in inoculated shoots at some of the measured times (**Figure 1**). While *AFB1* was up-regulated from 48 hpi, *AFB2* and *3* were down-regulated at 48 and 96 hpi, respectively. IAA1/AXR5 is involved in cell elongation as well as in cell division in the aerial parts of *Arabidopsis* plants and a role in vascular development has also been suggested (Ku et al., 2009). *IAA1/AXR5* gene was down and up-regulated in inoculated roots and shoots at 48 hpi, respectively (**Figure 2**). Interestingly, in the dominant mutant *axr5-1* (Yang et al., 2004), where there is deficient auxin response and a decrease in auxin-regulated transcription, none of the growth parameters were significantly increased when plants were inoculated with strain PsJN. This reveals that auxin perception through IAA1/AXR5 degradation is importantly involved in the plant growth stimulation induced by *B. phytofirmans* PsJN, affecting the outcomes of plant inoculation with this strain in the primary root growth, fresh weight and rosette area (**Figures 2** and **5**).

In plants, the ARF transcription factor family regulates gene expression in response to auxin repressing the Aux/IAA proteins. ARF7 regulates lateral root development, downstream regulating *PUCHI* (Jung and McCouch, 2013; Kazan, 2013). Strain PsJN up-regulated *ARF7* in roots (**Figure 2**), however, at least at the measured times, a difference in the lateral roots was not detected in the inoculated plants. Moreover, *PUCHI* was not regulated by PsJN inoculation (data not shown).

Recent evidence also points to a less well-known role for auxin as a mediator of environmental adaptation in plants (reviewed in Kazan, 2013). DRM1/ARP and DRM2/ARP are splicing variants of predicted intrinsically disordered nature belonging to a dormancy/auxin associated family protein. There is evidence relating these proteins with the response to sub-optimal biotic or abiotic conditions (Rae et al., 2014). *DRM2/ARP* was exceptionally up-regulated (more than 30 times) both in roots and shoots of inoculated plants at 96 hpi (**Figure 2**). Remarkably, this strain protects plants to several abiotic and biotic stresses (Ait Barka et al., 2006; Fernandez et al., 2012; Theocharis et al., 2012; Naveed et al., 2014; Pinedo et al., 2015; Su et al., 2015; Wang et al., 2016; Timmermann et al., unpublished). Additional analyses may evaluate a role for this protein in the beneficial effects of strain PsJN in plants under detrimental environmental conditions.

### Participation of Ethylene-Related Pathway in *Arabidopsis* Growth Promotion Induced by Strain PsJN

Together with auxin, ethylene also affects root growth by inhibiting the rapid expansion of cells leaving the root meristem (Růzicka et al., 2007; Swarup et al., 2007). Ethylene can regulate auxin biosynthesis and basipetal auxin transport toward the elongation zone, which activates auxin signaling in the root apex, and thus causes root growth inhibition (Růzicka et al., 2007; Stepanova et al., 2007; Swarup et al., 2007).

Several analyses of ethylene resistant mutants have identified components of auxin perception, *tir1* (Ruegger et al., 1998) and transport (*pin2/eir1* and *aux1*) (Pickett et al., 1990; Luschnig et al., 1998). The reduced sensitivity of *eir1* roots to inhibition by ethylene suggested that *EIR1* might be a gene involved in regulation of ethylene responses specifically in the root (Stepanova et al., 2007; Jung and McCouch, 2013). Interestingly, the most severe effects in PsJN inoculated *eir1-1* mutants were observed in roots, where the inoculation did not induce growth (**Figure 6**).

Ethylene is perceived by a family of ethylene response factors ETR1, ETR2, ERS1, ERS2, and EIN4 that are homologous to bacterial two-component His kinases (Shakeel et al., 2015). Upon ethylene binding, receptor inhibits the function of a Raf-like kinase, CONSTITUTIVE TRIPLE RESPONSE 1 (CTR1), allowing EIN2 to act as a positive regulator of the ethylene pathway (Ju et al., 2012). Recent findings have suggested that EIN2 controls final organ size by restricting cell expansion (Feng et al., 2015). *B. phytofirmans* PsJN did not induce plant growth in *ein2-1* mutants, but an increase in primary root length or rosette area was neither observed in the ethylene overproducer mutant *eto2.* The *eto2* mutant has an ACS5 protein with increased half-life (Chae et al., 2003). Correspondingly, this gene showed firstly an up-regulation in roots and then a down-regulation 96 hpi (**Figure 3**). These results indicate that a fine regulation of ethylene/ACC biosynthesis and response is needed for the growth promotion detected in PsJN inoculated plants, both in roots and aerial tissues. As *ACO1* and *2* genes were up-regulated only 2 hpi, an increment in ACC rather than in ethylene content after PsJN inoculation in roots cannot be discarded. ACC can be conjugated to three different derivatives with hitherto unclear biological roles and is subjected to a sophisticated transport mechanism to ensure local and long-distance ethylene responses (Van de Poel and Van Der Straeten, 2014). Also, Tsang et al. (2011) described an unconventional ACC-dependent mechanism that acts independently of ethylene signaling in the rapid inhibition of cell elongation triggered by cell wall stresses. These authors concluded that ACC *per se* has a short-term (few hours) influence on root cell elongation whereas, for long-term growth responses, the conversion of ACC to ethylene (ethylene-dependent pathway) is required (Tsang et al., 2011). Further investigations should dissect the role of ethylene and ACC in the changes of root architecture induced by this PGPR strain.

### Role of Auxin Transport and/or Ethylene Synthesis in Root Hair Development Under Strain PsJN Inoculation

Root hairs are essential for the uptake of water and nutrients, to facilitate interactions with soil microorganisms, and help anchor roots. Ethylene acts both synergistically and independently from auxin controlling the initiation, elongation, and cellular positioning of root hairs (Rahman et al., 2002). Although both auxin and ethylene positively regulate root hair formation, auxin is required for maximal root hair initiation and can overcome the reduced formation in the absence of ethylene. However, in root hair elongation and positioning, auxin and ethylene act synergistically, most likely through modulating cellular auxin concentration, with the two hormones having equivalent positive regulatory roles (Muday et al., 2012). Constitutive activation of ethylene signaling or treatment with exogenous ethylene or ACC promotes root hair elongation (Pitts et al., 1998). By contrast, the root hairs of ethylene-insensitive mutants and of seedlings treated with inhibitors of ethylene synthesis or signaling are significantly shorter (Rahman et al., 2002). Also, root hair elongation is enhanced by auxin treatment and in mutants that have elevated auxin synthesis, including *sur1* and a gain-of-function *yuc* allele (Boerjan et al., 1995; Pitts et al., 1998), while root hair development and elongation are reduced in auxin signaling mutants (Wilson et al., 1990; Leyser et al., 1996). Loss of ethylene signaling in the *ein2-1* mutant does not affect the ability of the roots to initiate root hairs. Nevertheless, when the intracellular auxin level is reduced by the application of auxin influx inhibitors (such as 1-naphthoxyacetic acid, 1-NOA), *ein2-1* shows a twofold greater inhibition of root hair initiation than untreated seedlings, and root hair initiation in wild type is reduced to the levels observed in untreated *aux1* mutant (Rahman et al., 2002), indicating that intracellular auxin and ethylene transduction pathways are fundamental for root hair development.

Strain PsJN augments root hair number and length in *Arabidopsis* (Poupin et al., 2013), regarding ethylene synthesis; *ACS5* was strongly induced in the early phases of PsJN inoculation and then was down-regulated (**Figure 3**). These results would suggest an enhancement of ACC and/or ethylene synthesis in *Arabidopsis* roots in the early stages of PsJN inoculation. When the inhibitor of ethylene synthesis AVG was used, root hairs were almost not detected, as described in Masucci and Schiefelbein (1994). Interestingly the same was observed when plants were also inoculated, indicating that ethylene synthesis in plants is essential for the PsJN-promoting root hair apparition. Instead, the auxin efflux inhibitor NPA alone significantly increases root hair number and length. But, remarkably an additive effect was observed in both parameters, when NPA treatment was combined with PsJN inoculation (**Figures 5A–C**). This allows the conclusion that strain PsJN augments root hair number/length through a mechanism that is not affected by NPA. Ljung et al. (2005) reported that in older seedlings treatment with NPA causes an accumulation of IAA in the root tip. It might be hypothesized that NPA augments auxin concentrations at the root tip and that this auxin can synergistically act with the ACC/ethylene produced after PsJN inoculation to increment root hair number and length. The auxin efflux mutant *eir1/pin2* has a short root hair phenotype, which has been attributed to a reduced auxin supply from the root tip to the hair differentiation zone (Cho et al., 2007). It has been reported that chemical inhibition of auxin transport with NPA do not impair the ethylene effect on root growth and cell elongation in comparison to that of *eir1* mutation (Růzicka et al., 2007), where *eir1-1* has ethylene resistance exclusively restricted to root growth (Roman et al., 1995; Stepanova et al., 2007). Interestingly, PsJN did not augment the root hair number but increased the hair length in *eir1-1* mutants (**Supplementary Figure S1**). A similar pattern was observed when *eto2* mutants were inoculated with strain PsJN (**Supplementary Figure S1**). A high root hair number was observed in non-inoculated *eto2* mutant plants and the inoculation did not increase this parameter but significantly augmented the length of root hairs. Together these results support the idea that ethylene homeostasis is a key component in the apparition of more root hairs under PsJN colonization.

Likewise under AVG treatment, root hairs were not detected when NPA was added together with AVG (**Figures 5A–C**). Since neither strain PsJN nor NPA individually overcomes the effect of AVG, the effect of AVG should be upstream of NPA or PsJN in root hair control. However, when plants were inoculated and treated with NPA concurrently, the effect of AVG was bypassed (**Figure 5**). This reinforces the idea that PsJN and NPA affect independent downstream pathways increasing the root hair number and length.

Previous studies have suggested that ethylene participates in the root architecture response to PGPR but is not a key player (reviewed in Vacheron et al., 2013). Interestingly, most of the studied PGPR such as *Pseudomonas fluorescens* WCS417, *P. fluorescens* WCS374, *P. putida* WCS358 (Zamioudis et al., 2013); *P. brassicacearum* (Contesto et al., 2008, 2010; Galland et al., 2012) and *B. megaterium* (Lopez-Bucio et al., 2007) have the opposite effect of strain PsJN, shortening the primary root and increasing the lateral root number. Here the role of ethylene and auxin signaling pathways in *Arabidopsis* growth induction mediated by strain PsJN was detailed and simultaneously studied, finding that both plant hormones are crucial for the beneficial effects of this PGPR. While auxin efflux transport is especially required to its role in the induction of primary root growth, an effective auxin signaling and perception through AXR5 is necessary for its growth-promoting effects, both in the aerial and roots tissues. Furthermore, ethylene signaling via EIN2 is also fundamental for growth induction mediated by this strain and the absence of growth promotion in some parameters in an ethylene overproducer (*eto2*) indicates that a fine tune regulation of ACC/ethylene contents are important for the beneficial effects of this interaction in plants. Finally, the roles of both hormones were studied in the PsJN-mediated promotion of root hairs growth, where it was found that ethylene biosynthesis plays a key role.

Overall, this study helps to understand the responses of plants under PGPR inoculation considering spatiotemporal transcriptional patterns and chemical and genetic approaches. These kind of approaches to study PGPR-plant interactions will not only allow us to better understand how these interactions occur and to understand the underlying mechanisms but also will give us useful hints to uncover signaling pathways involved in plant developmental processes.

## Author Contributions

MP is the corresponding author of the manuscript; she is responsible for conceiving the experimental strategies. She has collected and organized most of the relevant data, performed general interpretations of results, and has written and/or revised most of the text in the manuscript. He has coordinated general approval of the final version. MG, VC, and IP have performed most of the experiments shown throughout the manuscript, they contributed to experiment design and analysis of the results. Also they contributed to the revision of the general sections, and have given the approval of the final version.

## Conflict of Interest Statement

The authors declare that the research was conducted in the absence of any commercial or financial relationships that could be construed as a potential conflict of interest.

## Supplementary Material

**FIGURE S1 | Effects of *Burkholderia phytofirmans* PsJN in root hair number and length in the *eir1–1* and *eto2* mutants.** Graphic representation of the root hair number and hair length of root tips (5 mm) from *eir1–1* (A) and *eto2* mutants inoculated (PsJN) or not (Control) with *B. phytofirmans* PsJN. Data are means ± SE of at least 20 plants per treatment. Asterisk indicates statistical significance between the inoculated and non-inoculated plants in each genotype (Kruskal–Wallis non-parametric test, *p* < 0.05).

**TABLE S1 | List of real time RT-PCR primers.** Melting temperatures, amplicon sizes and references, if applicable, are indicated.
