# Supplementary material for: A Complex Molecular Interplay of Auxin and Ethylene Signaling Pathways Is Involved in Arabidopsis Growth Promotion by Burkholderia phytofirmans PsJN
Source: Front Plant Sci. 2016 Apr 12;7:492. doi: 10.3389/fpls.2016.00492 (PMC4828629; doi:10.3389/fpls.2016.00492)
Supplement: Supplementary file 2 [file Table_1.DOCX]

**Supplementary table 1:**

| **Locus (Name)** | **Primers (5-3`)** | **Tm (ºC)** | **Amplicon (bp)** | **Reference** |
| --- | --- | --- | --- | --- |
| SAND (AT2G28390) | F: AACTCTATGCAGCATTTGATCCACT  R: TGATTGCATATCTTTATCGCCATC | 59 | 61 | [1] |
| PIN2 (AT5G57090) | F: CCAATGTTCACGGGGTCAAC  R: GAAGCACTCGAACTCCACACG | 66 | 137 | Designed in the present study |
| PIN3 (AT1G70940) | F: CCCAGATCAATCTCACAACG  R: TTCTCCTCCGAAATCTCCAC | 60 | 70 | [2] |
| AFB1 (AT4G03190) | F: AAGCGAGTCTTTGTCGGAAACT R: CCCCTTCAAAGTCAAAGATCTCAT | 57 | 90 | [3] |
| AFB2 (AT3G26810) | F: CTGTGGTTGGGACAAGAATGG R: AACGGAAGACGACCAATCAGA | 57 | 93 | [3] |
| AFB3 (AT1G12820) | F: AGGAAGCTGGAGATAAGGGACAGT R: AAGGGATCGCATTGTTTCGT | 57 | 84 | [3] |
| ACO1 (AT2G19590) | F: TGGGTTCCTATACCGCCATC  R: ACCAGCCGGATTGTAAAACG | 60 | 162 | Designed in the present study |
| ACO2 (AT1G62380) | F: TGCAGGAGGCATCATCTTGT  R: TGCAACCGACATCCTGTTTC | 60 | 208 | Designed in the present study |
| TAA1 (AT1G70560) | F: CAAGTGGGAAGGAGACGCAT R: GGTTCACCACCGTCTCTCTG | 62 | 107 | Designed in the present study |
| ASA1 (AT5G05730) | F: GAGCCAACGAGGCGTGGACC  R: GGCTCGAGCAAGACCAGCGG | 58 | 258 | Designed in the present study |
| ASB1 (AT1G25220) | F: ACCACTCGCCGCCTAAACC  R: ATTGGACCATGCTGCTTAGAGG | 60 | 170 | Designed in the present study |
| IAA1 (AT4G14560) | F: TGGAAGTCACCAATGGGTTTAACCT R: TGCCGTTGTTGTTGCTTATGACG | 61 | 114 | Designed in the present study |
| PILS3 (AT1G76520) | F: GGAAGCTCCTCTCCGGGTGCT R: TAACGCGCAACCAAGACGCC | 61 | 156 | Designed in the present study |
| ACS5 (AT5G65800) | F: GGACTTCCTGGTTTCCGTGT R: GTCGGAGAGCAATGCAGAGA | 62 | 132 | Designed in the present study |
| ARF7 (AT5G20730) | F: TTTCTACAACCCGAGGGCTGCT R: ACCGCATACCGAGGGAAACTTGA | 65 | 95 | Designed in the present study |
| DRM2 (AT2G33830) | F: GATCGGCGCGTAAGGAAAAC R: CCATTCCTCTAGTGGCGATGTTA | 58 | 72 | Designed in the present study |

**REFERENCES**

1. Czechowski, T., Stitt, M., Altmann, T., Udvardi, M. K., and Scheible, W. R. 2005. Genome-wide identification and testing of superior reference genes for transcript normalization in Arabidopsis. Plant Physiol. 139:5- 17.
2. Lewis, D.R., Negi, S., Sukumar, P., and Muday, G.K. (2011). Ethylene inhibits lateral root development, increases IAA transport and expression of PIN3 and PIN7 auxin efflux carriers. Development 138, 3485-3495. doi: 10.1242/dev.065102.
3. Vidal, E. A., Araus, V., Lu, C., Parry, G., Green, P. J., Coruzzi, G. M., and Gutiérrez, R. A. 2010. Nitrate-responsive miR393/AFB3 regulatory module controls root system architecture in *Arabidopsis thaliana*. Proc. Natl. Acad. Sci. U.S.A. 107:4477-4482.
